# Mapping neural circuits with CLARITY

**DOI:** 10.7554/eLife.11409

**Published:** 2015-10-09

**Authors:** Amanda M Willard, Aryn H Gittis

**Affiliations:** Department of Biological Sciences and the Center for the Neural Basis of Cognition, Carnegie Mellon University, Pittsburgh, United States; Department of Biological Sciences and the Center for the Neural Basis of Cognition, Carnegie Mellon University, Pittsburgh, United Statesagittis@cmu.edu

**Keywords:** dopamine, rabies virus, striatum, anatomy, input, monosynaptic, mouse

## Abstract

The use of whole-brain imaging has shed new light on the organization of the dopamine system.

**Related research article** Menegas W, Bergan JF, Ogawa SK, Isogai Y, Umadevi Venkataraju K, Osten P, Uchida N, Watabe-Uchida M. 2015. Dopamine neurons projecting to the posterior striatum form an anatomically distinct subclass. *eLife* **4**:e10032. doi: 10.7554/eLife.10032

**Image** Rabies viruses labelled with fluorescent protein (green) can be used to map inputs and outputs for neurons in the dopamine system

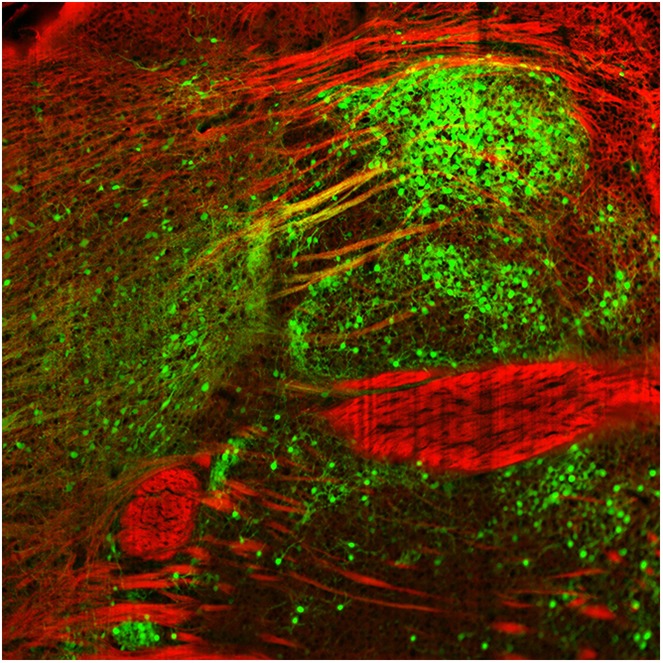

How can a relatively small number of neurons influence aspects of behavior as diverse as motivation, learning, reward and movement by releasing just one type of molecule? This is the challenge facing neuroscientists working on the dopamine system – a population of ∼30,000 neurons that are located in just two regions of the brain.

At first it was thought that the neurons in the dopamine system were all quite similar, but over the past decade it has become clear that they exhibit a range of molecular, anatomical and functional properties (Lammel et al., 2008; Margolis et al., 2008; Lammel et al., 2011). Moreover, it seems that distinct subsets of dopamine neurons belong to discrete circuits that carry out different functions, although the architecture of these circuits remains an open question. For the most part, projections from a given subset of dopamine neurons target one region of the brain (Yetnikoff et al., 2014), but projections to a given subset arise from many different regions. It has been challenging to determine whether the different subsets all receive the same inputs, or whether some regions of the brain project more to one subset than another.

Now, in eLife, Mitsuko Watabe-Uchida and colleagues at Harvard University and the Cold Spring Harbor Laboratory – including William Menegas as first author – report that almost all dopamine neurons receive the same set of inputs from a range of brain regions (Menegas et al., 2015). However, one exception is a subset of dopamine neurons that project to a region called the tail of the striatum (Figure 1).

**Figure 1.**
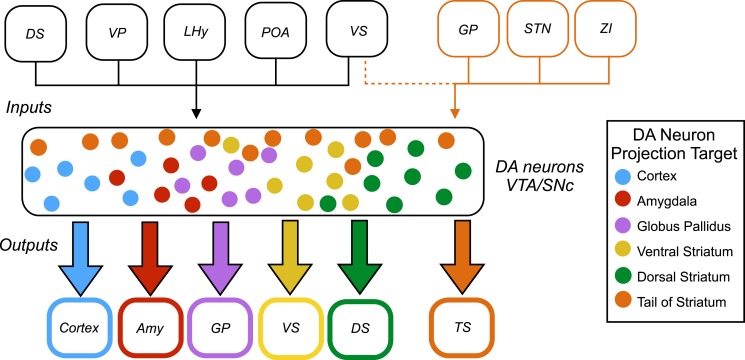
Inputs and outputs of dopamine neurons. Dopamine (DA) neurons are found in the ventral tegmental area (VTA) and the substantia nigra pars compacta (SNc). Dopamine neurons projecting to the tail of the striatum (TS) receive the majority of their input from the globus pallidus (GP), the subthalamic nucleus (STN), and the zona incerta (ZI), with a small amount of input coming from the ventral striatum (VS). Dopamine neurons that project to the cortex, amygdala (Amy), globus pallidus, ventral striatum and dorsal striatum (DS) receive the majority of their inputs from the following regions: the ventral striatum, dorsal striatum, ventral pallidum (VP), lateral hypothalamus (LHy), and preoptic area (POA). In Parkinson’s disease, dopamine neurons projecting to the posterior putamen (which is functionally similar to the tail of the striatum) are the first to degenerate (Kish et al., 1988), so their unique pattern of inputs is especially interesting to researchers.

Menegas et al. used a combination of a powerful anatomical technique called CLARITY (Chung et al., 2013) and light-sheet microscopy to map the input and output projections of dopamine neurons in an intact mouse brain. First, subsets of dopamine neurons were classified according to which of eight regions – medial pre-frontal cortex, orbitofrontal cortex, central amygdala, globus pallidus, ventral striatum, dorsal striatum, tail of the striatum, or lateral habenula – they projected onto. Next, a given subset, based on its projection target, was infected to express two proteins (avian retroviral receptor and rabies virus envelop glycoprotein). Then, three weeks later, a modified rabies virus was injected into the dopamine neurons. This virus spreads retrogradely and labels neurons projecting to the dopamine neurons with green fluorescent protein. Menegas et al. had to develop a suite of new data acquisition and analysis tools to map the 3D position of the fluorescently labeled neurons and align their results across animals.

This analysis revealed that most subsets of dopamine neurons receive similar inputs from a number of regions of the brain, primarily from the ventral striatum and the hypothalamus, regardless of projection target (Figure 1). This suggests that functional specialization in the dopamine system arises within the two regions where the dopamine neurons are found – the ventral tegmental area and the substantia nigra pars compacta – or downstream from these regions.

A notable exception was the subset of dopamine neurons that project to the tail of the striatum: the majority of the inputs to this subset came from regions of the brain that do not project strongly onto the other subsets (Figure 1). This suggests that the activity of this subset of neurons is regulated in a way that differs from the regulation of the other subsets of dopamine neurons.

The findings of Menegas et al. largely complement recent results on the organization of neural circuits within the dopamine system from two other groups, but a few differences are worth noting. Within the substantia nigra pars compacta, Lerner et al. observed a reciprocally connected architecture, with the neurons that project to the dorsomedial striatum receiving more input from this region, and likewise for the neurons that project to the dorsolateral striatum (Lerner et al., 2015). Additionally, Beier et al. observed a ‘biased input/discrete output’ architecture within the ventral tegmental area, with neurons projecting to the medial nucleus accumbens and the lateral nucleus accumbens receiving different patterns of input (Beier et al., 2015). These discrepancies may reflect some limitations of the automated analysis used by Menegas et al.

These results from Menegas et al. show that the outputs of dopaminergic neurons are more distinctive then their inputs. This has implications for how and where specialization of dopami-nergic circuits arises, and suggests that many important computations are performed locally within the ventral tegmental area and the substantia nigra pars compacta. An immediate challenge is to gain a deeper understanding of the local microcircuit dynamics that govern the activity of dopamine neurons within these two regions.
